# Seasonal Turnover and Functional Structure of the Foliar Mycobiota in a Gondwanan Temperate Forest Keystone Tree

**DOI:** 10.3390/jof11110795

**Published:** 2025-11-07

**Authors:** Lucía Molina, Mario Rajchenberg, María Belén Pildain, Mary Catherine Aime

**Affiliations:** 1 Fitopatología y Microbiología Aplicada, Centro de Investigación y Extensión Forestal Andino Patagónico (CIEFAP), Ruta 259 Km 16.24, Esquel 9200, Chubut, Argentina; 2 Consejo Nacional de Investigaciones Científicas y Técnicas (CONICET), Ciudad Autónoma de Buenos Aires C1425FQB, Argentina; 3 Facultad de Ciencias Naturales y Ciencias de la Salud, Universidad Nacional de la Patagonia San Juan Bosco (UNPSJB), Esquel 9200, Chubut, Argentina; 4 Department of Botany and Plant Pathology, Purdue University, West Lafayette, IN 47906, USA

**Keywords:** phyllosphere fungi, endophytes, high-throughput sequencing, functional guilds, temperate *Nothofagus* forests, *Taphrina entomospora*

## Abstract

Fungal communities inhabiting leaves are key players in ecosystem processes but remain largely unexplored in Southern Hemisphere temperate forests. We characterized the foliar mycobiota of *Nothofagus pumilio*, a dominant deciduous tree in Patagonian forests, using ITS1 metabarcoding across seasons and tree health conditions. We detected 426 fungal taxa, including a 40-Amplicon Sequence Variant (ASV) core mycobiome persisting year-round. Fungal richness and biomass increased significantly in autumn, coinciding with leaf senescence, and community composition shifted markedly between seasons. Spring leaves were enriched in pathogens and basidiomycetous yeasts, while autumn leaves hosted more saprotrophs, ascomycetous yeasts, and lichen-associated fungi. Tree health had limited influence on overall community structure, but symptomatic trees showed higher ASV richness and specific indicator taxa, including the pathogen *Trichosporiella multisporum* and members of the Taphrinaceae and Saccotheciaceae families. Despite taxonomic turnover, ecological guilds remained relatively stable, suggesting functional redundancy. These findings reveal a seasonal successional trajectory in the foliar mycobiota of *N. pumilio*, from early-colonizing endophytes in spring to diverse decomposer assemblages in autumn. This study provides the first high-throughput insight into the structure and dynamics of foliar fungal communities in Southern Hemisphere temperate forests, offering a baseline for understanding microbial roles in forest health and resilience.

## 1. Introduction

The phyllosphere—the aerial surfaces of plants—hosts one of the largest microbial habitats on Earth, playing a central role in plant–microbe–environment interactions [1,2]. Within this habitat, the foliar mycobiota encompasses diverse fungal lifestyles, including epiphytes, pathogens, saprobes, and especially endophytes—ubiquitous fungi that colonize internal leaf tissues asymptomatically and can influence host physiology, stress tolerance, and pathogen resistance [3,4,5]. These endophytes thrive in the selective microenvironment of the leaf interior and often engage in context-dependent interactions that span the mutualistic-pathogenicity continuum [4,6]. Despite their ecological relevance, studies have largely focused on crops or economically valuable species in tropical and Northern Hemisphere forests [7,8,9]. In contrast, fungal communities in long-lived native trees in regions such as South America remain largely unexplored [10]. Recent studies on plant-associated microbiota have emphasized the relevance of identifying the core mycobiome—the subset of fungal taxa consistently found across individuals or conditions—which may play central ecological roles in host functioning and microbial community stability [11,12,13].

Research on foliar endophytes in temperate forests has revealed their significant ecological functions, including contributions to plant stress tolerance, pathogen resistance, and nutrient acquisition [6]. Previous studies in Northern Hemisphere forests have reported seasonal shifts in foliar fungal communities. For instance, Osono [14] documented an increase in ligninolytic fungi during leaf senescence in *Fagus crenata*, suggesting a succession from endophytes to decomposers. Jumpponen and Jones [15] observed significant seasonal turnover in fungal endophytes of *Acer rubrum* deciduous leaves, with early colonizers replaced by different assemblages later in the growing season. Faticov et al. [16] reported that both fungal richness and community structure in leaf tissues of *Betula* spp. varied across seasons, strongly influenced by temperature and precipitation. However, natural forest ecosystems remain understudied compared to agricultural systems, and it is still unclear whether seasonal dynamics contribute substantially to temporal variability in long-lived tree species [9].

The genus *Nothofagus* is a key component of temperate forests across the Southern Hemisphere, often considered emblematic of Gondwanan biogeographical heritage due to its ancient origins and disjunct distribution in South America, Australasia, and Antarctica [17,18]. The Andean Patagonian forests represent one of the largest temperate forest reserves in the Southern Hemisphere and remain among the least anthropogenically disturbed globally [19]. Dominated by endemic *Nothofagus* species—which cover ~90% of the region [20]—these forests hold critical ecological and socio-economic value [21]. In northern Patagonia, *Nothofagus pumilio* prevails in high-elevation stands, including protected areas such as Los Alerces National Park, a UNESCO World Heritage site. However, these ecosystems face increasing threats from climate change, with reports of progressive and spatially clustered dieback in *N. pumilio*, particularly in its northernmost populations [22,23]. Although several fungal taxa have been isolated from symptomatic trees, no single pathogen has been confirmed as the causal agent [22]. Given the critical role of living trees as fungal reservoirs in temperate forests [24] and the ecological significance of plant-associated fungal communities in host health and forest biodiversity [25,26], characterizing the foliar mycobiota of *N. pumilio* is essential for understanding potential microbial contributions to forest resilience or decline.

Fungal communities inhabiting leaves are taxonomically and functionally diverse, typically dominated by members of the Ascomycota and Basidiomycota. Among ascomycetes, frequent foliar inhabitants include taxa within the orders Capnodiales, Pleosporales, and Helotiales, encompassing both endophytic and pathogenic lineages [27,28]. Basidiomycetes are also commonly detected, particularly in the form of yeasts and smut fungi, although their ecology remains less well characterized in many forest systems [25]. Yeasts, in particular, represent a key component of the phyllosphere in temperate forests. Though historically underrepresented, basidiomycetous yeasts such as *Solicoccozyma*, *Saitozyma*, and *Vishniacozyma* are frequently detected and may play disproportionate ecological roles due to their stress tolerance and functional plasticity [29,30].

Recent advances in high-throughput sequencing (HTS) and metabarcoding have enabled comprehensive profiling of plant-associated fungal communities, revealing strong influences of host traits, environmental filtering, and temporal variation on microbiota composition [5,7]. These methods have proven cost- and time-efficient for assessing microbial diversity at large scales with high sensitivity [31]. Within forest biomes, microbiome studies have mainly concentrated on tropical and subtropical wet forests, followed by temperate mixed forests, with a clear bias toward the Northern Hemisphere.

Research has documented the fungal diversity associated with Northern Patagonian forests, including polypores, ectomycorrhizal fungi, ophiostomatoid fungi, and decay fungi [32,33,34,35], but all these studies were associated with the stem and roots. Except for the description of the foliar pathogen *Thaprina entomospora* by Hansen et al. [36], there is no information on the Patagonian phyllosphere. Recently, we characterized the endophytic fungal communities of *N. pumilio* wood compartments in the context of grouped mortality using culture-dependent and culture-independent approaches [22,37,38]. Our findings revealed a rich and heterogeneous fungal diversity, strongly influenced by host-related factors such as health condition and plant compartment (roots and stems). Moreover, fungal communities in wood compartments exhibited higher susceptibility to seasonal and temperature variations. However, the fungal communities associated with *N. pumilio* leaves, which may play critical roles in tree physiology and responses to environmental stressors, remain largely unstudied. Given the pivotal role of fungi in forest health and functioning, understanding the diversity and distribution of foliar endophytes in native *Nothofagus* species is essential for assessing ecosystem stability under changing climatic conditions [10].

This study aims to comprehensively investigate the fungal communities associated with the leaves of *Nothofagus pumilio*, a key deciduous tree species native to Patagonia. Specifically, it seeks to identify and characterize the dominant fungal taxa, analyze their development throughout the growing season, and assess the impact of tree health conditions on these communities.

As a deciduous species, we hypothesize that fungal assemblages in spring will be smaller and more restricted, expanding as the growing season progresses and reaching their peak during senescence. Similarly, we expect symptomatic samples to resemble autumn assemblages, exhibiting higher diversity than those from healthy trees due to the weakened defense mechanisms, which may facilitate colonization by secondary taxa.

Regarding ecological guilds, we anticipate a higher prevalence of leaf pathogens in spring, targeting functional leaves, while in autumn, closer to senescence, we expect a more diverse assemblage of fungal guilds. In general, autumn samples should display a broader range of taxa and functional groups, including those involved in litter decomposition. In contrast, spring samples are expected to show a higher presence of yeasts, favored by the more optimal temperature conditions for their growth.

By addressing these aspects, this research provides valuable insights into the structure and dynamics of fungal communities in *N. pumilio* leaves, enhancing our understanding of their ecological significance.

## 2. Materials and Methods

### 2.1. Study Area and Sampling Procedure

**Figure 1:**
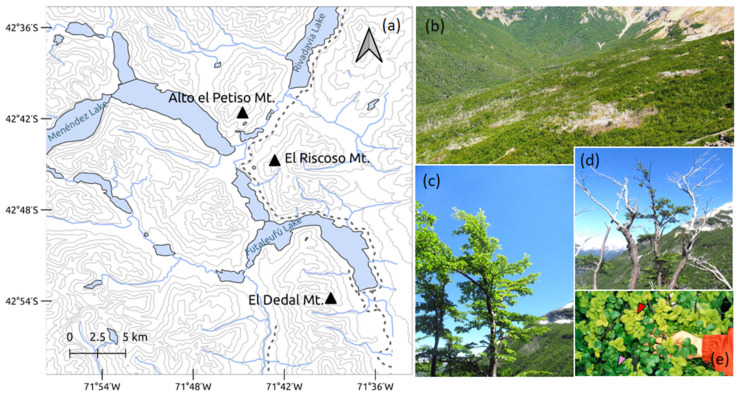
(**a**) Study area and sampling sites, with coordinates referenced to the WGS 84 geodetic system. (**b**) Grouped standing mortality of *Nothofagus pumilio* at El Dedal Mt. (Photo credit: Andrés de Errasti). (**c**) Asymptomatic, healthy-looking tree. (**d**) Symptomatic tree showing dead branches. (**e**) Leaves infected with *Taphrina entomospora* (red arrow) compared to healthy leaves (pink arrow).

The study took place in Los Alerces National Park (LANP) of Argentinian Patagonia (42°58′27.075″ S, 71°38′37.725″ W) between November 2017 and May 2018. Sampling was carried out seasonally at both the beginning and end of the growing season. Three *Nothofagus pumilio* stands were designated as sampling locations, all containing areas with standing tree mortality (Figure 1). The stands are located at an altitude of approximately 1300 m above sea level close to the tree line and receive an average annual precipitation of around 2000 mm [37].

For each sampling period and season, six to ten dominant trees were selected at each site, comprising three to five symptomatic and three to five asymptomatic individuals. The definition of symptomatology followed the criteria established by Molina et al. [22]. Briefly, trees in an early stage of decline were chosen from the edges of mortality patches, where the radial expansion of the patches was actively affecting new individuals. Symptomatic trees exhibited a transparent crown, dry branches, chlorosis, and defoliation affecting more than 25% of the canopy. In contrast, asymptomatic trees appeared healthy, with a crown cover ranging from 75% to 100%, and were located at least 80 m away from the edge of mortality patches (Figure 1). Within each site, selected trees were of comparable diameter at breast height (DBH). Each tree was sampled only once and a total of 46 trees were sampled during the study. In the Alto El Petiso Mount site, three symptomatic (St) and five asymptomatic (At) trees were sampled in spring, and four St and four At trees in autumn. In the El Dedal Mount site, four St and four At trees were sampled in both spring and autumn. In the El Riscoso Mount site, four St and four At trees were sampled in spring, and three St and three At trees in autumn. Spring samples correspond to the onset of leaf expansion and early physiological activity (mid-December), representing young, actively growing leaves, whereas autumn samples (mid-March) correspond to mature, fully expanded leaves collected before visible senescence, just prior to color change and leaf abscission. A composite leaf sample (about 10 leaves) was collected for each tree in sterile 2 mL Eppendorf tubes, which were surface-sterilized and homogenized following standardized methods described in Unterseher et al. [39] for assessing endophytic microbiota. Total DNA was isolated from homogenized samples using the DNeasy PowerPlant Pro Kit (QIAGEN, Hilden, Germany), following the manufacturer’s protocol. Libraries for the Internal Transcribed Spacer 1 (ITS1) region were prepared using a dual-indexing TruSeq (two-step) strategy as described in Molina et al. [37]. ITS1 amplification utilized the primer pair TS-ITS1-F (5′-ACACTCTTTCCCTACACGACGCTCTTCCGATCTCTTGGTCATTTAGAGGAAGTAA-3′) and TS-ITS2-R (5′-GTGACTGGAGTTCAGACGTGTGCTCTTCCGATCTGCTGCGTTCTTCATCGATGC-3′) [40,41]. Amplification reactions (25 μL total volume) were performed using MyTaq Mix (Bioline, London, UK) with the following thermocycling conditions: initial denaturation at 94 °C for 5 min; 32 cycles of 94 °C for 45 s, 50 °C for 45 s, and 72 °C for 1 min; and a final extension at 72 °C for 7 min.

PCR products were purified using ExoSAP-IT (USB Corporation, Cleveland, OH, USA) as per the manufacturer’s guidelines. Indexed libraries were generated using sample-specific barcode combinations with TruSeq primer pairs i5-TS-DI-5xx (AATGATACGGCGACCACCGAGATCTACAC 8-nucleotide barcode ACACTCTTTCCCTACACGAC) and i7-TS-DI-7xx (CAAGCAGAAGACGGCATACGAGAT 8-nucleotide barcode GTGACTGGAGTTCAGACGTG) (Integrated DNA Technologies, Redwood City, CA, USA). The indexing PCR involved an initial denaturation at 95 °C for 3 min; eight cycles of 95 °C for 30 s, 55 °C for 30 s, and 72 °C for 30 s; followed by a final extension at 72 °C for 5 min. The indexed products were again purified using ExoSAP-IT (Thermo Fisher Scientific, Waltham, MA, USA) as instructed by the manufacturer. DNA concentrations were measured with a NanoDrop spectrophotometer (ThermoFisher).

Negative controls from DNA extraction and PCR, as well as non-biological synthetic mock communities [42], were processed alongside the samples, amplified, included in the final pools, and sequenced. Libraries were pooled in equimolar concentrations and sequenced at the Purdue Genomics Core Facility (Purdue University, West Lafayette, IN, USA) using a MiSeq version 2 Reagent kit (500 cycles) on the Illumina MiSeq platform (2 × 250 bp).

### 2.2. Bioinformatic Analysis

The amplicon dataset was analyzed and processed using the Amplicon Toolkit (amptk) (version 1.2.4) [42], a tool demonstrated to be particularly effective for fungal ITS amplicon analysis [43,44]. The workflow began by trimming short reads [42] and removing primer sequences from the reads. Paired-end reads were then merged using usearch (version 9.2.64) [45], and the assembled reads were quality-filtered using the expected error trimming method [46].

Amplicon sequence variant (ASV) tables were generated using the dada2 pipeline (version 1.6.0) [47], applying a 97% identity threshold. Cross-contamination was addressed by identifying the sequences of the SynMock community and their frequencies within the dataset, allowing calculation of the tag-switching index [42]. Additionally, post-clustering curation was performed using the lulu algorithm (version 0.1.0) [48].

Taxonomic assignment of ASVs followed a “hybrid” method within the amptk platform, utilizing the UNITE database [49]. This approach integrated classifications based on global alignment, utax (RC Edgar, http://drive5.com/usearch/manual9.2/cmd_utax.html, accessed on 10 February 2025), and sintax [50]. The method selected the most reliable taxonomy, prioritizing the global alignment result (top hit) when the identity threshold exceeded 97%, or the classification with the highest confidence score from the other methods. In cases of conflicting taxonomies, the last common ancestor taxonomy was assigned [42].

Manual curation of the ASV tables was conducted in accordance with the guidelines provided by Brown et al. [51]. ASVs classified as non-fungal, undefined at the kingdom level, or represented by fewer than 10 reads were excluded from the final dataset. The taxonomy of ASVs underwent manual review and curation, with ecological guilds subsequently assigned. Each ASV sequence was queried against the GenBank database (https://blast.ncbi.nlm.nih.gov/, accessed on 5 November 2024) using the blastn algorithm [52]. Matches with an identity of 90–98% were classified at the genus level, while those exceeding 98% identity were assigned to the species level. In cases where conflicting taxonomies arose among multiple queried sequences, the last common ancestor taxonomy was designated. Finally, ecological guilds were assigned to the taxa by using the FunGuild tool (v.1.1).

### 2.3. Ecological Analysis

Manually curated ASVs were prepared in three different formats for downstream analyses. The read abundance table was used as a proxy for relative biomass, acknowledging the limitations inherent to sequencing data [42,53]. The data were also transformed to create a binary presence/absence table, representing ASV occurrences across samples. This approach is supported by the correction of ASV tables for tag-switching errors, which ensures confidence in the presence/absence data [51]. Lastly, a Hellinger-transformed table was generated, providing proportional values suitable for analyses based on relative abundance [54,55]. Community analyses were conducted using all of these data tables; when trends were equivalent, only the main selected results were presented to optimize the readability and focus of the manuscript.

The influence of season and health condition on LWIF richness was assessed at both the sample and variable levels using the Kruskal–Wallis rank-sum test [56] and Pearson’s Chi-square test [57], implemented with the “kruskal.test” and “chisq.test” functions from the stats R package version 4.1.2 [58].

Differences in community composition and structure across variable levels were evaluated with perMANOVA [59] using the “adonis” function in the vegan package (version 2.5.7) [55]. For abundance-based ASV tables, perMANOVA was applied to Bray–Curtis dissimilarity matrices [60] created with the “distance” function (method = “bray”). Hellinger-transformed matrices [54] were generated using the “decostand” function (method = “hellinger”) in vegan. For binary ASV tables, perMANOVA was conducted on Raup–Crick metric matrices [61] using the “raupcrick” function in vegan. Assumptions of multivariate homogeneity of dispersions were verified using the “betadisper” and “permutest” functions [62].

Community similarity patterns across seasons and health conditions were explored via non-metric multi-dimensional scaling (NMDS) ordinal plots [63] using the “ordinate” function (method = “nmds”) and visualized with “plot_ordination” in the phyloseq package (version 1.38.0) [64]. Venn diagrams illustrating shared and unique ASVs for variable levels were created with the eulerr package (version 6.1.1) [65].

Associations between fungal species and site groups were analyzed using a multilevel pattern analysis to identify indicator species, i.e., taxa significantly associated with particular habitat categories such as health condition (symptomatic or asymptomatic) or sampling seasons [66]. The significance of these associations was tested using permutation tests based on the IndVal method [67]. Indicator species analysis was performed using the ‘multipatt’ function from the indicspecies package (version 1.7.9) [66]. For the leaf ASV dataset, we use this analysis to detect species strongly associated with specific seasons and health conditions of the host.

PCR run and Illumina lane effects were accounted for in the statistical models to mitigate methodological biases. Data analysis and visualization were conducted in RStudio (version 4.1.2; http://www.rstudio.com/) using the aforementioned packages along with biomformat (version 1.22.0) [68] and ggplot2 (version 3.3.5) [69].

## 3. Results

### 3.1. Overall Diversity and Taxonomic Composition of the Foliar Mycobiota

**Figure 2:**
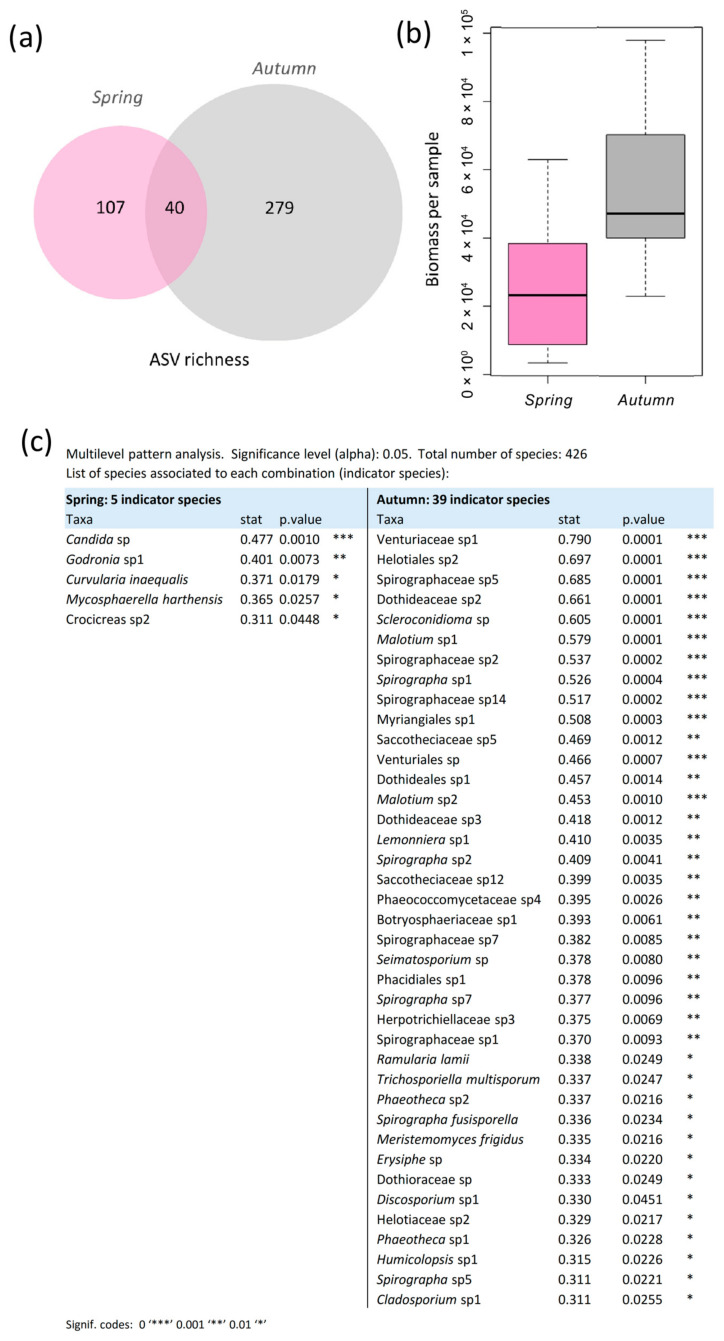
Assemblages across seasons. (**a**) Unique and shared ASVs across seasons. (**b**) Biomass per sample in spring and autumn. (**c**) Indicator species for spring and autumn detected by multilevel pattern analysis using the indicspecies package (version 1.7.9) in R.

The study identified 426 fungal taxa across 46 leaf samples (Table S1). ASV richness was higher in autumn (319 ASVs) than in spring (147 ASVs) (Figure 2). Additionally, fungal biomass per sample was significantly higher in autumn compared to spring (χ^2^ = 63.49, *p* < 0.001) (Figure 2).

**Figure 3:**
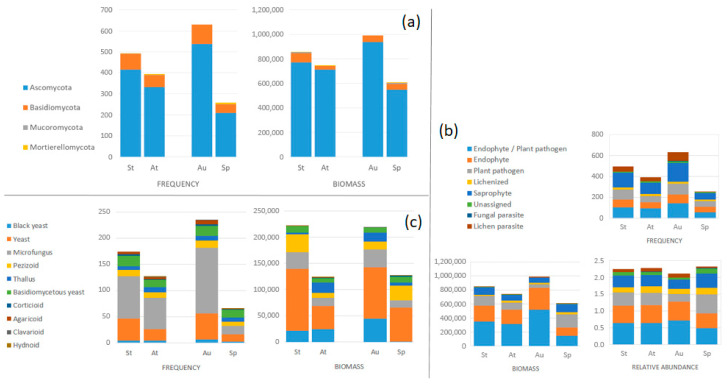
Frequency and biomass per health statuses and seasons. (**a**) Phylum. (**b**) Ecological guild. (**c**) Growth form. St: symptomatic. At: asymptomatic. Au: autumn. Sp: spring.

Leaf-associated fungal diversity was primarily composed of Ascomycota ASVs, followed by Basidiomycota ASVs (Figure 3). In contrast, only a few representatives of Mucoromycota and Mortierellomycota were detected, all of which were identified exclusively during the spring sampling.

### 3.2. Seasonal Effects on Fungal Richness, Biomass, and Community Structure

**Figure 4:**
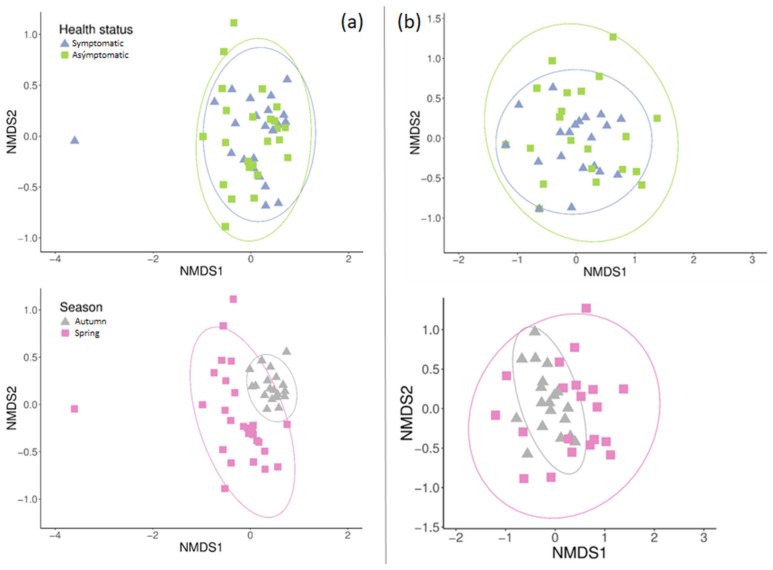
Community structure across health statuses (**above**) and seasons (**below**) based on non-metric multidimensional scaling (NMDS). (**a**) ASV structure. (**b**) Growth form structure. Ellipses indicate the dispersion of samples within each group at a 95% confidence level. Colors and shapes represent different health statuses and seasons.

The community structure of fungal assemblages varied significantly between seasons in terms of biomass, relative abundance, and ASV frequencies (Table 1, Figure 4). On the other hand, ANOSIM analyses indicated that health status did not affect community structure. Although fungal biomass and richness were higher in autumn than in spring, community structure exhibited lower dispersion in autumn when ordinated (Figure 4).

### 3.3. Seasonal Indicator Species and Core Mycobiome Composition

Multilevel pattern analysis identified a core of 39 indicator species associated with the autumn sampling while the spring was associated with five indicator species (Figure 2). Autumn indicators encompassed a broad range of genera, including *Cladosporium, Discosporium*, *Erysiphe*, *Humicolopsis*, *Lemonniera*, *Malotium*, *Meristemomyces*, *Phaeotheca*, *Scleroconidioma*, *Seimatosporium* and *Spirographa*. They also included representatives of several fungal families—Botryosphaeriaceae, Dothideaceae, Dothioraceae, Helotiaceae, Herpotrichiellaceae, Phaeococcomycetaceae, Saccotheciaceae, Spirographaceae and Venturiaceae—as well as taxa from the orders Helotiales, Myriangiales, Phacidiales and Venturiales. In contrast, spring indicators were fewer and included the genera *Candida*, *Godronia*, *Curvularia*, *Mycosphaerella*, and *Crocicreas*.

A total of 40 ASVs were shared between both seasons, forming the core mycobiome (Figure 2, Table 2). Among them are species that are abundant throughout the entire growing season, such as members of the family Venturiaceae; taxa that are abundant at the beginning of the season, such as a species of the genus *Crocicreas*, which decreases in abundance towards senescence; and, conversely, taxa that are rare at the beginning of the season but become abundant towards its end, such as several species of the genus *Spirographa*, a species of the genus *Scleroconidioma*, a species of the order Myriangiales, and members of the family Dothideaceae. Moreover, the core mycobiome also includes several yeast taxa, such as species of the genus *Lapidomyces*; members of the family Saccotheciaceae, closely related to *Aureobasidium* based on ITS sequence identity; taxa within the order Dothideales; the basidiomycetous family Kriegeriaceae; and the black yeast genus *Phaeotheca*. The core also includes various endophytic taxa (42%), and plant pathogens (42%) including several ASVs assigned to the family Taphrinaceae.

### 3.4. Seasonal Patterns in Ecological Guilds and Fungal Growth Forms

Ecological guild analyses revealed differences in guild structure across seasons in terms of frequency, relative abundance, and biomass (Table 1, Figure 4). Multilevel pattern analysis identified three guilds as indicators of the autumn season: endophyte/plant pathogen (Statistic = 0.704, *p* < 0.001), lichen parasite (Statistic = 0.522, *p* < 0.001), and endophyte (Statistic = 0.505, *p* < 0.001). No indicator guilds were found for spring or for the different health statuses.

The community structure of fungal growth forms was aggregated and found to differ between seasons (Table 1, Figure 4) in both relative abundance and frequency, according to PERMANOVA and ANOSIM analysis, respectively.

### 3.5. Influence of Tree Health Status on Fungal Community Composition

**Figure 5:**
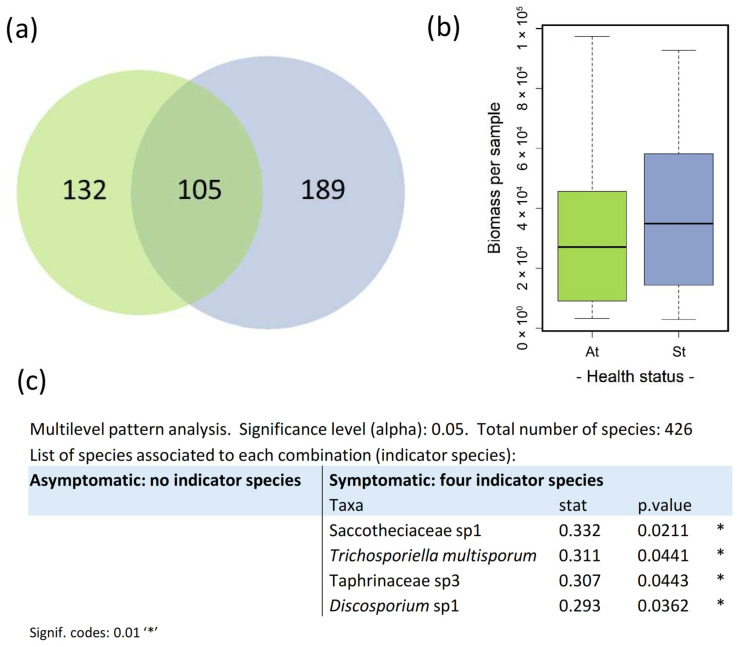
Assemblages across health statuses. (**a**) Unique and shared ASVs across health statuses: asymptomatic (green) and symptomatic (blue). (**b**) Biomass per sample in asymptomatic (At) and symptomatic (St) trees. (**c**) Indicator species detected by multilevel pattern analysis using the indicspecies package (version 1.7.9) in R.

Although biomass per sample in symptomatic trees was higher than in asymptomatic ones, the differences were not statistically significant. Symptomatic trees exhibited higher ASV richness (294) than healthy-looking ones (237) (χ^2^ = 6.12, *p* = 0.013) (Figure 5). Both health statuses share a core mycobiome of 105 ASVs. Multilevel pattern analysis did not find indicator species for asymptomatic trees, while it found four ASVs as indicators of symptomatic ones (Figure 5): a species from the Saccotheciaceae family (Dothideomycetes), the ascomycetous yeast *Trichosporiella multisporum*, a species from the pathogenic family Taphrinaceae (Taphrinomycetes), and a species from the pathogenic genus *Discosporium* (Sordariomycetes).

### 3.6. Yeast Community Structure and Seasonal Dynamics

**Figure 6:**
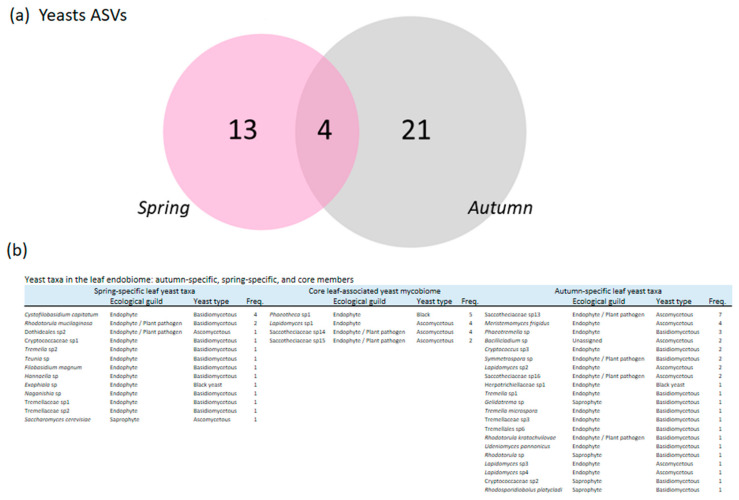
Seasonal variation in yeast assemblages. (**a**) Unique and shared yeast ASVs among seasons. (**b**) Taxonomic composition, ecological guilds, yeast types, and relative frequencies of spring-exclusive, autumn-exclusive, and core leaf-associated taxa.

Yeast communities were analyzed separately for each season (Figure 6). The community structure of ASVs with a yeast growth form did not differ between health statuses overall. However, in spring (at the beginning of the growing season for both the tree and its leaves), yeast community structure differed significantly between health statuses (F.Model = 2.206, R^2^ = 0.081, *p* = 0.001), exhibiting greater data dispersion among healthy-looking trees (Figure 5).

Basidiomycetous yeasts were consistently present across both seasons and health statuses, with a notable prevalence of *Tremella* and *Tremella*-like taxa, especially in autumn. Spring samples, by contrast, featured exclusive occurrences of species such as *Cystofilobasidium capitatum* and *Rhodotorula mucilaginosa*, both basidiomycetous yeasts commonly associated with endophytic lifestyles, although *R. mucilaginosa* is also known as a potential opportunistic plant pathogen. The core mycobiome included black yeasts such as a species of the genus *Phaeotheca* and several ascomycetous taxa including a species of genus *Lapidomyces* and members of the family Saccotheciaceae, many of which are putative endophytes or have mixed roles as endophytes and pathogens. Other ascomycetous yeasts showed greater variability between seasons and tree health status, being more abundant and frequent in autumn and in symptomatic trees than in healthy individuals or spring samples (Table 1, Figure 5 and Figure 6). This group included taxa such as *Meristemomyces frigidus*, predominantly endophytes, a species of the family Saccotheciaceae, and a species of the basidiomycetous genus *Phaeotremella*. Ascomycetous yeasts represented the dominant fungal biomass in *N. pumilio* leaves (Figure 5 and Figure 6).

## 4. Discussion

To the best of our knowledge, this is the first study to investigate the foliar-associated fungal communities of temperate forests in the Southern Hemisphere using a culture-independent, high-throughput sequencing approach. Despite the ecological importance of these ecosystems—particularly those dominated by *Nothofagus* species, the most abundant and widely distributed tree genus in the region—research on phyllosphere fungal communities in Southern Hemisphere forests remains extremely limited. To our knowledge, there is only one published study addressing foliar fungal communities in temperate forests of the Southern Hemisphere [70]. That study focused on a different genus within the family Nothofagaceae and relied on the pyrosequencing technology.

This study provides novel insights into the fungal communities associated with *Nothofagus pumilio* leaves, revealing significant seasonal and health-related variations in fungal diversity, biomass, and community structure at both the taxonomic and functional levels, including guilds and growth forms. Our approach followed a well-established metabarcoding framework focused on community composition and seasonality, providing a solid baseline for future integrative analyses that incorporate physiological and nutritional leaf traits.

### 4.1. Fungal Lineages and Their Seasonal Ecological Roles

Our findings highlight the dominance of Ascomycota, followed by Basidiomycota, as the primary fungal phyla present in *N. pumilio* foliage, consistent with previous studies on foliar mycobiomes in temperate forests [71,72,73]. This pattern may reflect several ecological traits of ascomycetous fungi, including their tolerance to the highly variable conditions of the phyllosphere—such as frequent desiccation, intense UV radiation, and temperature fluctuations [1,74]—as well as their ability to persist as latent endophytes or opportunistic saprotrophs [4]. Their efficient aerial dispersal and capacity to produce diverse secondary metabolites may further contribute to their competitive success in colonizing and maintaining foliar niches [3,4].

In contrast, Basidiomycota, although present, tend to occur in lower abundance. This could be due to their generally slower growth rates, which limit competitive ability in dynamic leaf-foliage environments [75], and to their narrower ecological tolerances or higher niche-specialization compared to Ascomycota [76], such as wood decomposition or ectomycorrhizal associations [77]. Some basidiomycetes may function as transient colonizers or become more prevalent during leaf senescence or after tissue damage [14].

*Nothofagus pumilio*, the dominant deciduous tree in sub-Antarctic forests of southern South America, is characterized by leaves with relatively short lifespans and high concentrations of phenolic compounds, including tannins [78,79]. These traits may influence fungal colonization by selecting for taxa adapted to chemically defended, ephemeral substrates. Moreover, the pronounced seasonality of *N. pumilio* forests, including leaf abscission and overwintering under snow, may further structure foliar fungal communities by favoring taxa capable of rapid colonization or seasonal cycling.

The observed leaf mycobiota in this study also found a limited presence of Mucoromycota and Mortierellomycota, which were exclusively detected in the spring samples. This seasonal occurrence could be related to the life history strategies and ecological roles of these early-diverging fungal lineages. Members of Mucoromycota and Mortierellomycota are often fast-growing, opportunistic saprotrophs that may colonize fresh leaf surfaces during early phenological stages, when plant tissues are still developing and nutrient leakage is higher [77,80]. Additionally, their transient detection might reflect a seasonal pulse in spore dispersal or environmental conditions—such as increased moisture and moderate temperatures—that favor their establishment during spring [21,81]. Interestingly, in a previous study on *Nothofagus pumilio* [37], these same phyla were detected in the stem and root endospheres, where we discussed their potential role as systemic endophytes. This raises the possibility that they may colonize the foliar tissues via vertical transmission during the onset of the growing season in this deciduous species, only to be later displaced through ecological succession by fungal assemblages with higher competitive or colonization abilities. Such a pattern would be consistent with a dynamic phyllosphere where early-season colonizers are gradually replaced by more specialized or better-adapted taxa as leaves mature and environmental conditions shift.

This trend is also observed in Basidiomycota ASVs, which increase in frequency during autumn, towards the end of the growing season and as the leaves approach senescence. This pattern is consistent with their role in leaf decomposition, particularly during the later stages of organic matter breakdown [82]. Basidiomycota fungi are well known for their lignin-degrading abilities, making them key players in the decomposition of woody and leaf litter [83]. However, as discussed below, there is an important portion of basidiomycetous ASVs that are yeasts.

### 4.2. Seasonality Shapes Fungal Community Structure

The average temperature during spring sampling in the stands is 7.9 °C, with a mean monthly precipitation of 52 mm. In contrast, during autumn, the average temperature is 3.2 °C and the mean monthly precipitation increases to 131 mm [37]. The results of this study reveal a clear seasonal variation in the fungal community present in leaf samples, with a significantly higher richness and biomass of fungal taxa observed in autumn compared to spring. The increased ASV richness in autumn, with 319 ASVs compared to 147 in spring, aligns with previous studies suggesting that fungal communities can be more diverse and abundant during cooler, wetter months [13,84]. This is consistent with the general ecological understanding that autumn provides more favorable conditions for fungal growth, including higher humidity, which likely support a more diverse fungal assemblage [25].

Seasonal differences strongly influenced fungal richness, biomass, and guild composition. The higher ASV richness and fungal biomass detected in autumn suggest an accumulation of fungal propagules and increased colonization as leaves mature and senesce. This pattern is consistent with findings in other deciduous tree species, where foliar fungal communities tend to diversify over the growing season, often reaching their peak just before leaf abscission [82]. Notably, among the autumn indicator species, we identified a taxon of the powdery mildew genus *Erysiphe*, likely *Erysiphe nothofagi*—the only species known to occur in these forests, although it currently lacks a representative sequence in public databases. Species of *Erysiphe* are commonly associated with the late stages of leaf development and are frequently observed near or during leaf senescence, as reported in other deciduous hosts such as *Quercus robur* [85]. Similarly, species of fungi in the family Botryosphareaceae, known for their endophytic and latent pathogenic lifestyles, are often observed in aging or stressed plant tissues, where they can switch from endophytism to saprophytism [86]. The detection of *Seimatosporium* sp.—a genus with necrotrophic tendencies—may also reflect its capacity to exploit senescent or decaying tissues [87]. Additionally, the presence of taxa such as *Scleroconidioma* sp. and *Ramularia lamii*, which have been described in association with declining or decaying foliage [88], supports the idea of a seasonal shift toward assemblages composed of species adapted to senescent tissues. These findings are consistent with ecological succession in the phyllosphere, where early colonizers are gradually replaced by fungi better suited for resource exploitation in aging leaves [82]. In addition, several other fungal taxa identified as autumn indicators may reflect a shift towards communities adapted to senescing tissues and early stages of litter decomposition. Members of the Helotiales, for instance, encompass a wide array of saprotrophic species frequently isolated from decomposing leaves and other plant debris [82]. Within this order, the family Helotiaceae has been particularly associated with early colonization of leaf litter, with some species capable of establishing in attached, senescent leaves and persisting after abscission [14]. Similarly, the detection of species in the Phacidiales in autumn samples may suggest the presence of fungi with ecological roles as litter colonizers. Although this order is less well characterized, several studies have reported its members in decomposing foliar material [89]. The species *Trichosporiella multisporum*, while not extensively studied, belongs to a lineage of Ascomycota with known endophytic habits and saprotrophic capabilities, suggesting its potential role in late-stage foliar colonization. Moreover, *Cladosporium* is a ubiquitous genus also in the Ascomycota, often associated with aging or stressed plant tissues. While commonly considered a phylloplane inhabitant, certain *Cladosporium* species show increased abundance during leaf senescence, likely taking advantage of weakened host defenses and nutrient leakage [90]. *Discosporium* is a genus of coelomycetous fungi that can exhibit saprobic or parasitic lifestyles on terrestrial plants, suggesting its potential role in colonizing senescent or decaying leaves [91,92]. Taken together, the presence of these taxa in autumn samples suggests the emergence of fungal guilds pre-adapted to exploit the structural and chemical changes in senescing leaves, either through early litter colonization or the ability to persist and proliferate as decomposition proceeds.

In contrast to the richer and more compositionally coherent fungal assemblages observed in autumn, the indicator analysis for spring yielded significantly fewer taxa with high indicator values. This pattern is consistent with the lower fungal richness and biomass detected in spring samples and reflects a higher compositional heterogeneity, as also evidenced by the broad dispersion of spring samples in the NMDS ordination. Such variability may arise from the stochastic colonization dynamics typical of early-season microbial communities, when environmental conditions are less stable and resource availability is more limited or patchy [93,94,95]. Despite the low number of indicator taxa, the species detected in spring may reveal insights into the functional strategies employed during early leaf development and in colder conditions. The yeast *Candida* sp., for instance, is a common inhabitant of the phyllosphere, often dominant under moist, low-UV environments typical of early spring [29]. Its ability to rapidly colonize and proliferate on young leaf surfaces suggests a strategy adapted to early successional stages and nutrient-rich exudates from developing tissues. *Godronia* sp., while less well studied, belongs to the Helotiales and includes species associated with overwintering structures or early colonization of leaf and stem tissues, sometimes as endophytes or latent pathogens [89]. Its presence in spring may reflect activity during the reactivation of host metabolism or the decomposition of overwintered material. Although classic monographs do not describe this genus in Patagonia [96], there is an undescribed species of *Godronia* found as a pathogen of living leaves of another Patagonian tree species: *Lomatia ferruginea* (Rajchenberg M. pers. comm.). *Curvularia inaequalis* is a dematiaceous fungus with a wide ecological amplitude, frequently isolated from warm-temperate and subtropical leaves. It can behave as both an endophyte and a saprobe [97], with some reports of early colonization of fresh litter or living foliage under stress. Its spring association might relate to its capacity for rapid growth and exploitation of ephemeral resources. *Mycosphaerella harthensis* is also known as a foliar endophyte in temperate deciduous trees and has been reported to sporulate on living or recently senescent leaves [98]. Its early detection might suggest either latent colonization prior to visible senescence or the utilization of early-season leaf damage. Finally, *Crocicreas* sp., another member of the Helotiales, is generally associated with cold-adapted environments and has been isolated from decaying leaves and bark in alpine or subalpine systems [99]. Its presence in spring may reflect tolerance to low temperatures and the ability to colonize plant tissues in early decomposition stages or during late snowmelt. Collectively, these taxa do not reflect a cohesive functional guild, but rather suggest a mixture of ecological strategies adapted to early-season environmental filters—such as cold tolerance, opportunistic colonization, and affinity for young or physiologically stressed host tissues. Notably, many of these fungi include potential pathogens, which may exploit early developmental stages of the leaves when defenses are not fully established. The high dispersion of fungal communities in spring supports the interpretation that assembly is driven more by stochastic events and transient environmental windows than by consistent, host-mediated selection.

The core mycobiome, comprising 40 ASVs shared between both seasons, likely includes fungal taxa with endophytic or latent saprotrophic lifestyles that persist throughout leaf development. Several fungal taxa exhibited consistent occurrence patterns across all seasons and treatments, suggesting potential systemic behavior within the host. Taxa belonging to the genera *Lapidomyces*, *Claussenomyces*, and *Rachicladosporium* were detected under all conditions, indicating a stable endophytic presence, possibly mutualistic or commensal in nature [27,100]. Notably, these three genera are melanized fungi which have been found inhabiting rock surfaces in Antarctica [101], exhibiting the tendency to survive in extremely cold environments. Melanin is also an adaptation that protects against UV rays [102,103], which is a common restraint in high altitudes and latitudes. In contrast, other genera showed marked seasonal fluctuations, likely reflecting successional dynamics within the endophytic community. For instance, a member of the family Venturiaceae and a species of the genus *Scleroconidioma* increased during autumn, whereas species from the genera *Crocicreas* and *Cistella* were more abundant in spring—patterns potentially driven by host phenology or the availability of senescent tissues [14,104].

### 4.3. Impact of Tree Health on Fungal Assemblages

Among the core mycobiome, certain taxa were predominantly associated with symptomatic plants, including members of the families Saccotheciaceae and Dothideaceae, suggesting a possible pathogenic role or enhanced proliferation under host stress conditions [105]. Among the core mycobiome a species from the family Herpotrichiellaceae was exclusively found in asymptomatic plants—pointing to a potential protective function or a preference for non-altered tissues. Conversely, taxa assigned to the family Taphrinaceae and the genus *Taphrina*, likely corresponding to *Taphrina entomospora*, were detected only in symptomatic individuals. This is consistent with their known foliar pathogenic behavior, as *T. entomospora* is the only described species of *Taphrina* occurring in temperate forests of the Southern Hemisphere and is a well-documented biotrophic pathogen infecting *Nothofagus* species (*N. pumilio* and *N. antartica*) [36]. Notably, no sequences of this species are currently available in public databases, which entirely precludes its taxonomic assignment through metabarcoding, despite morphological and ecological consistency with the observed patterns.

Contrary to expectations, health status did not significantly affect overall fungal community structure, as indicated by ANOSIM analyses. However, symptomatic trees exhibited significantly higher ASV richness, possibly due to increased colonization by opportunistic fungi [27]. The identification of fungal indicators associated with symptomatic trees—such as *Trichosporiella multisporum* and species from the Saccotheciaceae and Taphrinaceae families—suggests that certain fungi may act as pathogens or secondary colonizers of stressed hosts [104]. In contrast, no ecological guilds were significantly associated with tree health, implying that fungal functional roles may not shift drastically in response to host conditions —unlike what was previously observed in this species’ wood compartments, where fungal guild structure varied with host health status [37,106].

Among the indicator species detected in symptomatic trees were members of the family Saccotheciaceae, primarily known as saprobes colonizing senescent or decaying plant tissues, particularly in leaves and bark, and contributing to decomposition and nutrient cycling in forest ecosystems [107]. Similarly, *Trichosporiella multisporum*, an anamorphic ascomycetous yeast often isolated from soil and decomposing plant matter, may increase in prevalence under host stress conditions, potentially benefiting from weakened defenses or altered leaf microenvironments [108]. Species of the genus *Discosporium* are saprotrophic or weakly pathogenic fungi associated with woody tissues and leaf litter. Their elevated abundance in symptomatic hosts may reflect early decay processes or latent pathogenicity triggered by stress [91]. Consistently, taxa assigned to the family Taphrinaceae and the genus *Taphrina* were exclusively detected in symptomatic individuals; this case is discussed above. Taken together, the presence of these fungal taxa in symptomatic trees suggests that multiple fungal guilds—ranging from obligate pathogens to opportunistic saprotrophs—may act synergistically or sequentially in the progression of foliar decline. These interactions may be modulated by host stressors such as drought, mechanical injury, or insect activity, which can increase tissue susceptibility and facilitate fungal colonization [84,109].

The finding that symptomatic trees exhibited higher ASV richness suggests a potential link between fungal diversity and plant health. This may indicate that fungal communities in symptomatic trees are more diverse due to increased microbial activity associated with plant stress or disease [110].

### 4.4. Yeast Dynamics and Their Functional Role

Yeasts, as a functionally and phylogenetically diverse group within the foliar mycobiome, have been recognized for their roles in early colonization, nutrient competition, and potential antagonism against filamentous fungi and pathogens [29]. In this study, although the overall structure of yeast communities did not differ significantly between symptomatic and asymptomatic trees across all samples, a notable seasonal effect emerged. Specifically, at the beginning of the growing season (spring), yeast community structure diverged significantly according to host health status, with greater multivariate dispersion among healthy-looking trees. This greater dispersion—or increased beta diversity—among asymptomatic individuals may reflect higher inter-individual variability in yeast assemblages under less stressful conditions, possibly driven by more stochastic colonization processes [111]. In contrast, symptomatic trees might host more homogeneous yeast communities due to environmental filtering, where stress-related factors such as reduced leaf integrity or altered chemical profiles constrain the establishment to a narrower subset of yeast taxa [112,113]. These patterns align with the stress-gradient hypothesis, which posits that under increasing stress, community assembly becomes more deterministic due to abiotic filtering, thereby reducing compositional variability [114].

Additionally, the onset of leaf development in spring may be a critical window for yeast establishment, particularly on young, nutrient-rich tissues that present unique microhabitats. In this context, early colonizers might exert priority effects that shape downstream microbial interactions [115]. The observed differences in yeast community structure suggest that the initial phyllosphere colonization is influenced not only by environmental conditions but also by the physiological state of the host, which could modulate microbial recruitment and compatibility. These findings warrant further exploration of yeast ecological roles in foliar environments, especially considering that some yeast taxa can function as biocontrol agents or modulate plant stress responses [116,117]. Understanding how host health influences these early microbial dynamics could provide insights into the microbial determinants of resilience or decline in forest trees.

The analysis of seasonal patterns in the leaf endophytic mycobiome of *N. pumilio* reveals a marked taxonomic turnover of yeast species between spring and autumn. While both seasons exhibit high frequencies of basidiomycetous yeasts, the identities of these taxa are largely distinct, suggesting functional continuity coupled with taxonomic replacement. In spring, dominant taxa included *Cystofilobasidium capitatum*, *Rhodotorula mucilaginosa*, and members of Cryptococcaceae and *Tremella* species, while autumn was characterized by different representatives species of the genera *Cryptococcus*, *Symmetrospora*, and *Phaeotremella*. From a functional perspective, many of the yeasts identified in both spring and autumn belong to ecological guilds associated with endophytism and occasional plant pathogenicity. This suggests that functional redundancy may buffer the mycobiome against seasonal environmental variability, even as species identities change.

*Rhodotorula mucilaginosa*, for example, is a cosmopolitan yeast often associated with early successional stages of microbial colonization on plant surfaces [118]. Its detection in spring suggests a capacity to exploit newly emerged leaf tissues. Similarly, *Cystofilobasidium* spp. are known for their psychrotolerance and may benefit from the cooler spring temperatures in the Patagonian forest understory [119,120].

In contrast, the autumn-specific community includes several ascomycetous yeasts such as *Meristemomyces frigidus*, *Bacillicladium* sp., and multiple *Saccotheciaceae* spp., along with basidiomycetous genera like *Phaeotremella* and *Symmetrospora*. These taxa are typically associated with more advanced stages of leaf development or senescence, where increased cell wall degradation and altered moisture conditions favor the establishment of stress-tolerant or saprotrophic species [90,121]. *Phaeotremella*, for instance, includes species capable of parasitizing other fungi or persisting in decaying tissues—strategies potentially advantageous in late-season foliage [122,123].

Of particular note is the taxonomic diversity within the family Saccotheciaceae, which includes multiple ASVs found exclusively in autumn. Although the precise ecological roles of these lineages remain unclear, several are recurrently detected in senescent leaves and decaying plant material, suggesting a facultative saprotrophic strategy [10].

The presence of certain taxa with putative plant pathogenic potential, such as some Dothideales and *Symmetrospora* spp., raises the possibility that latent infections or opportunistic colonization may play a role in the autumnal phase, when host defenses may be reduced. However, the dual assignment of many of these taxa to both endophytic and pathogenic guilds underscores the fluidity of fungal lifestyles, which are often context-dependent [4,6].

## 5. Conclusions

Our study reveals that foliar fungal communities associated with *Nothofagus pumilio* exhibit marked seasonal dynamics in Patagonian temperate forests, with a strong taxonomic turnover—particularly among yeasts—yet a notable functional continuity across ecological guilds. These dynamics are shaped by environmental filtering, host phenology, and niche specialization, leading to temporally structured but ecologically resilient mycobiota.

Autumn emerges as a season of fungal richness and functional expansion. This phase is characterized by the highest ASV richness and fungal biomass, a broad set of indicator species, and a core community with taxa well adapted to senescent or declining tissues. The increased presence of saprotrophs, latent pathogens, and lichen parasites suggests a transition from endophytic colonization to early-stage decomposition, highlighting autumn as a critical window for phyllosphere community turnover and ecosystem-level nutrient cycling. Notably, key autumn indicator taxa—including *Trichosporiella multisporum*, *Discosporium* spp., and members of Taphrinaceae—are associated with symptomatic hosts, suggesting that tree health may modulate the expression of fungal lifestyles within these dynamic communities.

In contrast, spring hosts a more stochastic assemblage of fungi, with lower richness, biomass, and fewer indicator species. This season likely reflects early colonization events under colder, more variable conditions and less mature host tissue. However, yeasts—especially basidiomycetous taxa—are prevalent in spring, possibly facilitated by moderate microclimatic conditions and nutrient-rich young leaves. These early-season yeasts may establish priority effects, shaping subsequent community assembly.

Despite strong seasonal shifts in taxonomic composition, the ecological guild structure remains relatively stable, dominated year-round by endophytes and facultative saprotrophs. This functional redundancy suggests that while species identities shift, the ecosystem roles filled by foliar fungi are maintained—a hallmark of resilience in microbial communities.

Interestingly, although tree health status did not significantly alter overall community structure, symptomatic trees exhibited higher ASV richness and hosted a distinct set of indicator species. This may reflect increased niche availability due to physiological stress or compromised defenses. Still, the consistent detection of a shared core mycobiome—comprising 40 ASVs across seasons and 105 across health conditions—points to a subset of generalist taxa adapted to the *N. pumilio* foliar environment, including melanized endophytes and cold-adapted yeasts, with potential protective or mutualistic roles.

Yeast assemblages, in particular, underscore the dynamic equilibrium between taxonomic turnover and functional continuity. Basidiomycetous yeasts persist across seasons, while ascomycetous taxa show pronounced shifts, increasing in autumn and in symptomatic trees. This indicates that yeast diversity, phenology, and host health interact to shape colonization patterns, potentially influencing broader phyllosphere processes.

Altogether, these findings highlight the importance of integrating taxonomic, functional, and seasonal perspectives to understand the assembly and ecological roles of foliar fungal communities. In *Nothofagus pumilio*, the phyllosphere is not only a reservoir of hidden fungal diversity but also a dynamic interface where environmental, phenological, and health-related filters drive successional processes. This work contributes a foundational framework for studying fungal biodiversity and host–microbe interactions in temperate deciduous forests of the Southern Hemisphere.

## Figures and Tables

**Table 1:** Inferential analyses of fungal community structure of *N. pumilio* leaves across seasons and health statuses.

Amplicon Sequence Variant (ASV) Structure ^1^							
	Biomass		Relative abundance		ASV frequency		
	Statistic R	*p*-value	Statistic R	*p*-value	Statistic R	*p*-value	
Health status	0.023	0.173	0.031	0.141	0.021	0.202	
Season	0.332	0.001 ***	0.402	0.001 ***	0.392	0.001 ***	
Ecological guild structure							
	Biomass ^1^		Relative abundance ^1^		ASV frequency ^2^		
	Statistic R	*p*-value	Statistic R	*p*-value	Statistic F	R^2^	*p*-value
Health status	0.008	0.526	0.018	0.754	0.836	0.015	0.514
Season	0.258	0.001 ***	0.201	0.001 ***	10.771	0.197	0.001 ***
Growth form structure							
	Biomass ^1^		Relative abundance ^2^			ASV frequency ^1^	
	Statistic R	*p*-value	Statistic F	R^2^	*p*-value	Statistic R	*p*-value
Health status	0.038	0.104	0.670	0.057	0.658	0.016	0.651
Season	0.016	0.309	2.652	0.215	0.017 *	0.074	0.036 *

*p*-values: *** ≤ 0.001 ≤ * ≤ 0.05 ≤ ^1^ ANOSIM. ^2^ PERMANOVA.

**Table 2:** Core mycobiome from *N. pumilo* leaves. Taxa frequencies across health statuses and seasons. Taxa names are presented in alphabetical order.

	St	At	Sp	Au	Ecological Guild	Growth Form
*Alpinaria* sp	2	3	1	4	Plant pathogen	
Botryosphaeriaceae sp1	7	3	2	8	Saprophyte	
Chrysozymaceae sp	0	3	1	2	Unassigned	
*Cistella* sp1	4	2	5	1	Saprophyte	
*Cladophialophora* sp1	3	2	4	1	Saprophyte	
*Claussenomyces* sp	1	1	1	1	Saprophyte	
Clavariaceae sp	3	3	4	2	Lichenized	
*Crocicreas* sp2	5	4	8	1	Saprophyte	Pezizoid
*Discosporium* sp1	5	1	1	5	Plant pathogen	Microfungus
Dothideaceae sp3	4	4	1	7	Endophyte/Plant pathogen	
Dothideaceae sp4	7	2	3	6	Endophyte/Plant pathogen	
Dothideales sp3	1	1	1	1	Endophyte/Plant pathogen	Yeast
Dothideales sp4	3	1	2	2	Endophyte/Plant pathogen	Yeast
*Godronia* sp2	2	4	4	2	Plant pathogen	
Herpotrichiellaceae sp2	0	2	1	1	Saprophyte	
*Heterocephalacria* sp2	5	1	2	4	Saprophyte	
*Hyaloscypha* sp	2	1	2	1	Plant pathogen	
Kriegeriaceae sp1	3	0	1	2	Plant pathogen	Basidiomycetous Yeast
*Lapidomyces* sp1	2	2	2	2	Endophyte	Yeast
*Malotium* sp4	0	2	1	1	Saprophyte	Microfungus
*Meristemomyces* sp1	2	1	1	2	Endophyte	
Mycosphaerellaceae sp1	1	2	1	2	Plant pathogen	
Myriangiales sp1	4	7	1	10	Endophyte/Plant pathogen	
*Neophaeomoniella eucalyptigena*	2	1	2	1	Plant pathogen	
*Oidiodendron* sp *	2	2	3	1	Endophyte	
Phaeococcomycetaceae sp1	1	1	1	1	Endophyte	
*Phaeotheca* sp1	3	2	1	4	Endophyte	Black Yeast
*Piskurozyma* sp1	1	1	1	1	Saprophyte	
*Pleospora* sp	1	2	1	2	Endophyte/Plant pathogen	
*Rachicladosporium* sp	1	1	1	1	Endophyte	
Saccharomycetes sp1	2	0	1	1	Saprophyte	
Saccotheciaceae sp1	9	1	5	5	Endophyte/Plant pathogen	Yeast
*Scleroconidioma* sp	6	10	3	13	Endophyte	
*Spirographa* sp1	5	6	1	10	Lichen parasite	Microfungus
*Spirographa* sp2	3	5	1	7	Lichen parasite	Microfungus
Taphrinaceae sp1	1	1	1	1	Plant pathogen	Rust/Yeast
Taphrinaceae sp3	4	0	1	3	Plant pathogen	Rust
*Trichoderma* sp	2	0	1	1	Endophyte	
Venturiaceae sp1	12	14	8	18	Endophyte/Plant pathogen	
*Vishniacozyma victoriae*	1	1	1	1	Endophyte	

St: symptomatic. At: asymptomatic. Au: autumn. Sp: spring. * Insect associated.

## Data Availability

Raw sequence reads are deposited in the Short Read Archive of the National Center for Biotechnology Information (BioProject ID: PRJNA785007).

## Supplementary Materials

The following supporting information can be downloaded at: https://www.mdpi.com/article/10.3390/jof11110795/s1, Table S1: Taxa frequency for each site, health status and season.
